# Cost-effectiveness analysis of trastuzumab deruxtecan versus ramucirumab plus paclitaxel as second-line treatment for HER2-positive metastatic gastric cancer or gastroesophageal junction adenocarcinoma

**DOI:** 10.3389/fphar.2026.1811077

**Published:** 2026-06-25

**Authors:** Yitian Lang, Jie Yang, Min Liu, Yan Lin

**Affiliations:** 1 Department of Pharmacy, Shanghai Second People’s Hospital, Shanghai, China; 2 Department of Pharmacy, Tongren Hospital, Shanghai Jiao Tong University School of Medicine, Shanghai, China

**Keywords:** Cost-Effectiveness, gastric cancer, gastroesophageal junction adenocarcinoma, partitioned survival approach, Ramucirumab plus Paclitaxel regimen, trastuzumab deruxtecan

## Abstract

**Background:**

Trastuzumab Deruxtecan (T-DXd) regimen has demonstrated substantial antitumor activity in the treatment of HER2-positive Metastatic Gastric Cancer (GC) or Gastroesophageal junction adenocarcinoma (GEJA). However, the cost-effectiveness of this regimen in this patient population remains uncertain, particularly within the Chinese healthcare system context.

**Objective:**

This study aimed to assess the cost-effectiveness of T-DXd regimen compared with ramucirumab plus paclitaxel regimen as second-line treatment for patients with HER2-positive advanced GC or GEJA, from the perspective of the Chinese healthcare system.

**Methods:**

A partitioned survival model was developed to project the disease progression. Data for overall survival (OS) and progression-free survival (PFS) were extracted from the DESTINY-Gastric04 trial and were extrapolated to project long-term survival outcomes. Direct medical costs and utility values were gathered. The main outcome measures, including the cost, utility, and incremental cost-utility ratio (ICUR), were used to determine the cost-effectiveness of the T-DXd regimen. Sensitivity analyses, including one-way sensitivity analysis (OWSA) and probabilistic sensitivity analysis (PSA), were performed to evaluate the robustness of the findings.

**Results:**

The base-case analysis revealed that the ICUR for T-DXd regimen was ¥$607,788.5 per quality-adjusted life year (QALY) compared to the ramucirumab plus paclitaxel regimen. OWSA showed that the ICUR was sensitive to the cost of subsequent treatment, weight and unit price of the two drugs. Results from the PSA indicated that T-DXd regimen had a probability of cost-effectiveness, at 4.3%. In contrast, ramucirumab plus paclitaxel regimen demonstrated a high likelihood of being cost-effective, with a 95.7% probability.

**Conclusion:**

In conclusion, from the perspective of the Chinese healthcare system, T-DXd was not cost-effective compared with the ramucirumab plus paclitaxel regimen for HER2-positive metastatic GC or GEJA under trial-based post-progression treatment patterns. However, when subsequent therapy in both arms is standardized to a common regimen and costs are limited to ¥28,053 per cycle, T-DXd will become the more cost-effective option. When only pre-progression outcomes were considered, T-DXd was associated with lower costs and greater survival benefits.

## Introduction

Gastric cancer (GC) poses a substantial disease burden and remains one of the leading causes of cancer-associated mortality on a global scale. According to the latest GLOBOCAN 2022 estimates, approximately 968,000 newly diagnosed cases and 659,000 stomach cancer-related deaths were reported, making it the fifth leading cause of cancer-related deaths worldwide (Bray et al., 2024). Gastric cancer still remains a major health burden worldwide, with a notably high disease burden in East Asia (Bray et al., 2024). Due to demographic characteristics, dietary habits, and insufficient early screening coverage, China accounts for nearly half of gastric cancer-related deaths worldwide, with reporting 358,672 new cases and 260,372 deaths from gastric cancer in 2022 (Chen et al., 2016; International Agency for Research on Cancer, 2024).

For advanced GC and gastroesophageal junction adenocarcinoma (GEJA), treatment selection is guided by tumor subtype and includes chemotherapy, targeted agents, and immune checkpoint inhibitors. Among these subtypes, human epidermal growth factor receptor 2 (HER2) overexpression or amplification is observed in 7%–34% of advanced GC or GEJA cases and is associated with more aggressive tumor behavior and poorer prognosis in several studies (Wang et al., 2026; Gravalos and Jimeno, 2008; Smyth et al., 2020).

The treatment landscape for HER2-positive metastatic GC/GEJA has evolved substantially over the past decade. First-line therapy is firmly established as trastuzumab, a monoclonal antibody targeting HER2, combined with fluoropyrimidine-based chemotherapy, based on the landmark ToGA trial which demonstrated improved overall survival (OS) compared to chemotherapy alone (Bang et al., 2010). Despite the survival benefits achieved with trastuzumab-based first-line therapy, most patients eventually develop resistance to HER2-targeted agents. Inevitably, disease progresses in most patients, while traditional second-line treatment options remain limited, with modest survival benefits. This creates an unmet clinical need for effective subsequent-line treatment strategies and optimal healthcare resource allocation (Smyth et al., 2020; Pietrantonio et al., 2016). Ramucirumab (a vascular endothelial growth factor receptor 2 inhibitor) plus paclitaxel has been the standard second-line regimen since the RAINBOW trial, which showed a median OS of 9.6 months and established its role regardless of HER2 status (Wilke et al., 2014). Despite this, the unmet need for more effective second-line therapies remains high, as Ramucirumab plus Paclitaxel fails to address the HER2-driven oncogenic pathway, leading to suboptimal response rates and limited durability.

Trastuzumab deruxtecan (T-DXd), an antibody-drug conjugate (ADC) consisting of a humanized anti-HER2 monoclonal antibody linked to a topoisomerase I inhibitor payload, has demonstrated meaningful clinical activity in HER2-positive malignancies (Bang et al., 2010; Shitara et al., 2019). Based on findings from the DESTINY-Gastric01, 02, and 06 trials, T-DXd has been approved in multiple countries for second-line or later treatment of HER2-positive advanced GC/GEJA patients who have progressed on trastuzumab-based therapy (Shitara et al., 2020; Van Cutsem et al., 2023; Peng et al., 2025). The phase 3 DESTINY-Gastric04 trial further confirmed its superiority over ramucirumab plus paclitaxel, demonstrating a median OS of 14.7 months versus 11.4 months (hazard ratio [HR] = 0.70; 95% CI, 0.55–0.90; P = 0.004) and a median progression-free survival (PFS) of 6.7 months versus 5.6 months (HR = 0.74; 95% CI, 0.59–0.92; P = 0.007) (Shitara et al., 2025). With the emergence of T-DXd and other HER2-targeted agents, the sequencing of HER2-directed therapies has become increasingly important in advanced GC/GEJA. In clinical practice, patients receiving standard second-line therapy may subsequently receive T-DXd in later-line settings, whereas patients treated with T-DXd earlier may receive antiangiogenic or chemotherapy-based regimens after progression. These evolving treatment pathways may substantially influence long-term economic outcomes, particularly in resource-constrained healthcare systems such as China. In China, the National Healthcare Security Administration faces the dual challenge of expanding access to innovative anticancer drugs while maintaining the sustainability of the basic medical insurance system. With population aging and rising cancer incidence, healthcare expenditure on oncology therapies is growing rapidly. High-cost agents such as T-DXd pose significant financial pressure on both patients and payers. Drug reimbursement decisions in China increasingly rely on health economic evidence, which integrates clinical efficacy, safety, and treatment costs to balance therapeutic value and budget impact. However, to date, no study has yet evaluated the cost-effectiveness of T-DXd versus ramucirumab plus paclitaxel from the Chinese healthcare system perspective, using head-to-head phase 3 trial data. Existing economic evaluations of GC therapies have focused on first-line treatments or non-HER2-targeted agents, leaving a critical gap in evidence for second-line HER2-positive disease.

Therefore, this study aims to conduct a cost-effectiveness analysis of T-DXd versus ramucirumab plus paclitaxel as second-line therapy for HER2-positive advanced GC/GEJA from the Chinese healthcare system perspective, using data from the DESTINY-Gastric04 trial. The findings of this study will provide critical evidence for decision-makers, clinicians, and patients, supporting evidence-based resource allocation and improving access to high-value anticancer therapies in China.

## Materials and methods

The Consolidated Health Economic Evaluation Reporting Standards (CHEERS) serves as a comprehensive framework to enhance the reproducibility and transparency of health economic evaluations. The current economic evaluation rigorously followed the CHEERS 2022 guidelines, as detailed in Supplementary Table 1 (Husereau et al., 2013; Husereau et al., 2022).

**TABLE 1:** Key clinical data.

Parameters	Estimated values	Range	Distribution
PFS: T-DXd group	meanlog = 1.9646	meanlog (1.8487–2.0805)	Log-normal
	sdlog = 0.8286	sdlog (0.7421–0.9253)	
OS: T-DXd group	shape = 1.782	shape (1.541–2.062)	Log-logistic
	scale = 15.828	scale (13.745–18.227)	
PFS: Ramucirumab plus Paclitaxel group	shape = 2.099 scale = 5.661	shape (1.849–2.382) scale (5.044–6.355)	Log-logistic
OS: Ramucirumab plus Paclitaxel group	shape = 1.3841 scale = 16.7342	shape (1.2166–1.5746) scale (14.8575–18.8479)	Weibull
Risk of main grade 3 and more AEs in the T-DXd group			
Neutropenia	28.7%	21.53%–35.88%	Beta
Anemia	13.9%	10.43%–17.38%	Beta
Leukopenia	7.4%	5.55%–9.25%	Beta
Thrombocytopenia	8.6%	6.45%–10.75%	Beta
Risk of main grade 3 and more AEs in the Ramucirumab plus Paclitaxel group			
Neutropenia	35.6%	26.7%–44.5%	Beta
Anemia	13.7%	10.28%–17.13%	Beta
Leukopenia	12.4%	9.3%–15.5%	Beta
Thrombocytopenia	3%	2.25%–3.75%	Beta

*Abbreviations: T-DXd*, Trastuzumab deruxtecan; *AE*, adverse event; *PFS*, progression-free survival; *OS*, overall survival; *NA* not applicable.

### Model structure

**FIGURE 1:**
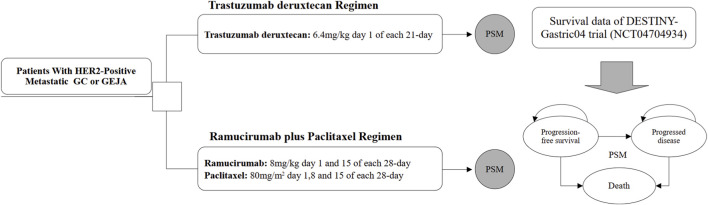
The decision tree and partitioned survival model structure overview. **Abbreviations: *GC*** Gastric cancer, ***GEJA*** gastroesophageal junction adenocarcinoma, ***PSM*** partitioned survival model, ***HER2*** human epidermal growth factor receptor-2.

A decision-analytic framework was developed to evaluate the cost-effectiveness of T-DXd regimen compared with the ramucirumab plus paclitaxel regimen in patients with metastatic GC or GEJA, from the perspective of the Chinese healthcare system. Disease progression was simulated using a partitioned survival model (PSM), which is recommended by National Institute for Health and Care Excellence (NICE) and commonly applied in oncology economic evaluations (Beth et al., 2017). The simulated patient cohort consisted of individuals with T-DXd regimen versus ramucirumab plus paclitaxel regimen, in accordance with the criteria used in the DESTINY-Gastric04 trial (Shitara et al., 2025). The PSM comprised three mutually exclusive health states: PFS, progressed disease (PD), and death. All patients entered the model in the PFS state and were allowed to transition to the PD or death according to survival probabilities derived from clinical trial data. The proportions of patients occupying each health state at each model cycle were estimated using independently fitted parametric functions for PFS and OS. A model cycle length of 4 weeks was adopted to simplify cost estimation, and a ten-year time horizon was applied to ensure that high proportion of patients transitioned to the death state, thereby capturing long-term clinical and economic outcomes. The overall model structure and decision pathways are illustrated in Figure 1.

### Clinical data

The current analysis relied on data from the DESTINY-Gastric04 trial, which reported detailed PFS, OS, and safety outcomes (Shitara et al., 2025). Owing to the relatively short follow-up period of the trial, direct use of the observed survival data was insufficient for long-term economic modeling, and extrapolation beyond the trial horizon was therefore required. Parametric survival modeling typically relies on access to individual patient data (IPD). In the absence of publicly available IPD, pseudo-individual patient data were reconstructed from the published Kaplan–Meier curves using the method developed by Guyot et al. (2012). This method has gained widespread acceptance in survival analysis research for generating reliable pseudo-IPD (Saluja et al., 2019). The reconstructed datasets were subsequently fitted to a range of candidate parametric distributions, including Log-logistic, Weibull, exponential, Log-normal and Gompertz distribution (Jackson, 2016). Model selection was guided by the Akaike Information Criterion (AIC), Bayesian Information Criterion (BIC), and visual inspection of fit. Survival data used in this analysis were obtained from the survival data provided in the DESTINY-Gastric04 trial.

In addition to survival outcomes, treatment-related adverse events (AEs) were incorporated into the analysis. Grade 1 and 2 AEs are usually mild in symptoms and thus generally well manageable in routine clinical practice (Trotti et al., 2003). Therefore, the analysis focused on grade 3 or higher AEs with an incidence exceeding 5%. A summary of the survival model parameters and AE incidence rates is presented in Table 1.

### Treatment regimens and resource use

In this model, the simulated patient cohort was allocated to two treatment arms based on the treatment regimen: the T-DXd arm and ramucirumab plus paclitaxel arm. Drug dosing and administration schedules for both regimens followed the recommendations of the CSCO Clinical Guidelines (Chinese Society of Clinical Oncology, 2025). T-DXd was administrated intravenously at a dose of 6.4 mg/kg every 21-day. In the comparator arm, paclitaxel was given intravenously at 80 mg/m^2^ of body surface area (BSA) on day 1, 8 and 15 of each 28-day, while ramucirumab was administrated intravenously at a dose of 8 mg/kg on day 1 and 15 of each 28-day. Because the DESTINY-Gastric04 trial did not explicitly define the maximum duration of treatment exposure or subsequent therapy, treatment duration in the model was dynamically estimated according to survival-state occupancy within the PSM.

In both treatment arms, therapy was continued until disease progression, unacceptable toxicity. The posttrial anticancer therapy regimens were implemented in accordance with the regimens reported by the DESTINY-Gastric04 trial and presented in Supplementary Table S2. Given the diversity of subsequent treatment options, we observed that ramucirumab plus paclitaxel was the most frequently administered subsequent therapy in the T-DXd group, whereas T-DXd was the most commonly used subsequent therapy in the ramucirumab plus paclitaxel group. To reduce model complexity, these two regimens were therefore assumed to represent the subsequent third-line treatment strategies in the cost-effectiveness analysis.

### Costs and utilities

The economic evaluation was performed from the perspective of the Chinese healthcare system and incorporated only direct medical costs. These costs covered expenditures for anticancer drugs, intravenous drug administration, follow-up, palliative care, the subsequent treatment cost and the management of AEs of grade 3 or higher. Follow-up costs comprised routine imaging examinations, diagnostic procedures, and disease monitoring during treatment. Drug acquisition prices were obtained from a local pricing database (Chinese Drug Price of Drug Centralized Bid Procurement, 2026), while costs related to intravenous administration, palliative care, follow-up visits were extracted from published literature (Lang et al., 2023; Zhang et al., 2023). The subsequent treatment cost parameters, reflecting the weighted average per-cycle cost of post-progression management strategies, including third-line T-DXd and ramucirumab plus paclitaxel regimens, were estimated based on the DESTINY-Gastric04 trial (Shitara et al., 2025). Management expenses for severe AEs graded as level 3 or higher were also derived from published studies. Costs associated with the management of severe AEs were also extracted from previously published studies (Lang et al., 2025; Zhu J. et al., 2025).

Health utility values were incorporated into the cost-effectiveness model to estimate total quality-adjusted life-years (QALYs), which reflect patients’ health-related quality of life (HRQoL) over the disease course. Distinct utility values were assigned to each health state, including PFS and PD. In this analysis, utility estimates were sourced from the RAINBOW trial, which reported HRQoL outcomes for patients receiving ramucirumab plus paclitaxel as second-line therapy for GC or GEJA (Wilke et al., 2014). Both cost and utility outcomes were discounted at an annual rate of 5% to reflect the time value of money and quality of life adjustments. Detailed model input parameters are presented in Table 2.

**TABLE 2:** Key model inputs Costs, Utility estimates and other parameters.

Parameter	Distribution	Values (range)	References
Treatment costs			
Trastuzumab Deruxtecan (per 100 mg)	Gamma	¥3,480 (2,610 to 4,350)	(Chinese Drug Price of Drug Centralized Bid Procurement, 2026)
Ramucirumab (per 500 mg)	Gamma	¥14,999.05 (11,249.29 to 18,748.81)	(Chinese Drug Price of Drug Centralized Bid Procurement, 2026)
Paclitaxel (per 30 mg)	Gamma	¥37.88 (28.41–47.35)	(Chinese Drug Price of Drug Centralized Bid Procurement, 2026)
Subsequent treatment for T-DXd group (per cycle)	Gamma	¥32,778.22 (24,583.67 to 40,972.78)	Estimated based on post-trial treatment regimens from the DESTINY-Gastric04 trial
Subsequent treatment for ramucirumab plus paclitaxel group (per cycle)	Gamma	¥19,955.71 (14,966.78 to 24,944.64)	Estimated based on post-trial treatment regimens from the DESTINY-Gastric04 trial
Administration (per cycle)	Gamma	¥532.01 (399.01–665.01)	Lang et al. (2025)
Follow-up	Gamma	¥332.25 (249.19–415.31)	Lang et al. (2025)
palliative care	Gamma	¥13,570.81 (10,178.11 to 16,963.51)	Lang et al. (2025)
AEs unit costs			
Neutropenia	Gamma	¥3,406.36 (2,554.77 to 4,257.95)	Lang et al. (2025)
Anemia	Gamma	¥1,059.14 (794.36–1,323.93)	Zhu J. et al. (2025)
Leukopenia	Gamma	¥3,572.53 (2,679.40 to 4,465.67)	Zhu J. et al. (2025)
Thrombocytopenia	Gamma	¥11,496.13 (8,622.10 to 14,370.16)	Zhu J. et al. (2025)
Utility estimates			
PFS state	Beta	0.75 (0.726–0.774)	Wilke et al. (2014)
PD state	Beta	0.61 (0.567–0.653)	Wilke et al. (2014)
Disutility estimates			
Neutropenia	Beta	0.20 (0.15–0.25)	Lang et al. (2025)
Anemia	Beta	0.073 (0.055–0.091)	Zhu J. et al. (2025)
Leukopenia	Beta	0.20 (0.15–0.25)	Zhu J. et al. (2025)
Thrombocytopenia	Beta	0.09 (0.068–0.113)	Zhu J. et al. (2025)
Other parameters			
Weight	Normal	67.20 kg (50.4–84)	Estimated based on sex ratio reported in the DESTINY-Gastric04 trial and reference (National Physical Fitness Monitoring Center, 2026)
Body surface area	Normal	1.73 m^2^ (1.30–2.16)	Estimated based on sex ratio reported in the DESTINY-Gastric04 trial and reference (National Physical Fitness Monitoring Center, 2026)

In this table, the costs of AEs, presented were paid on a per-event basis.

**Abbreviations: *AEs*** adverse events, ***T-DXd*** Trastuzumab Deruxtecan, ***PFS*** progression-free survival, ***PD*** progressed disease.

### Analyses

A base-case analysis was conducted to evaluate the cost-effectiveness of the two treatment regimens. The incremental cost - effectiveness ratio (ICER) was calculated to estimate the additional cost per life - year (LY) gained when comparing the two treatment regimens. Additionally, the incremental cost-utility ratio (ICUR) was used to assess the incremental cost per quality-adjusted life-year (QALY). A treatment strategy was defined as cost-effective if its ICUR was below the established willingness-to-pay (WTP) threshold. In this analysis, the WTP threshold was set at three times the *per capita* gross domestic product (GDP). For the year 2025, this threshold was estimated to 298,995 Chinese Yuan (CNY), which was applied as the reference threshold for determining cost-effectiveness from the Chinese healthcare system perspective.

Uncertainty surrounding the model parameters was evaluated through both one-way deterministic sensitivity analysis (DSA) and probabilistic sensitivity analysis (PSA). DSA was performed to examine the impact of uncertainty in individual model parameters on the ICUR. The annual discount rate was varied from 0% to 8%, while other parameters were adjusted within their reported 95% confidence intervals or, when unavailable, within a plausible range defined as ±25% of the base-case values. For the PSA, Monte Carlo simulations with 10,000 iterations were performed, allowing key parameters to vary simultaneously according to predefined probability distributions. Gamma distributions were used for cost parameters because costs are positive and right-skewed variables, whereas Beta distributions were assigned to utility values and AE probabilities because these parameters are constrained between 0 and 1 (Briggs et al., 2012). Cost-effectiveness acceptability curves (CEACs) and incremental cost-effectiveness scatter plots were generated to illustrate the probability of the T-DXd regimen being cost-effective across a range of WTP thresholds. All analyses were conducted using R software version 4.4.2.

## Results

### Curve fitting

Based on a combination of Akaike Information Criterion (AIC), Bayesian Information Criterion (BIC), and visual inspection of goodness of fit, the log-logistic distribution was selected to extrapolate OS for patients in the T-DXd group, while the log-normal distribution provided the best fit for PFS in the same group. In the ramucirumab plus paclitaxel group, OS was best characterized by a Weibull distribution, whereas PFS was modeled using a log-logistic distribution.

**FIGURE 2:**
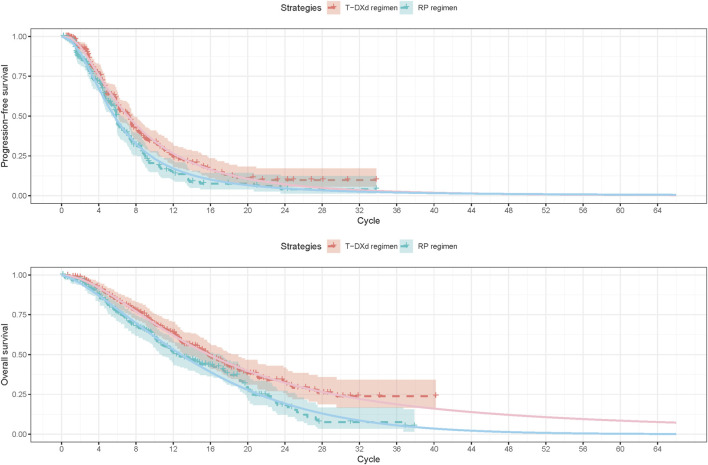
Reconstructed Kaplan-Meier survival curve and the projected OS and PFS curve. Abbreviations: ***T-DXd*** Trastuzumab Deruxtecan, ***RP*** ramucirumab plus paclitaxel. Notes: Each cycle of the x-axis is 4 weeks.

Using the selected parametric functions, replicated Kaplan–Meier curves were generated, and long-term PFS and OS trajectories were projected to facilitate comparison between the T-DXd regimen and the ramucirumab plus paclitaxel regimen, as illustrated in Figure 2. The corresponding scale and shape parameters for the fitted survival curves of both treatment strategies are reported in Table 1. Results from alternative parametric distributions, along with their respective fitted curves, are presented in Supplementary Figure S1, and detailed parameter estimates for these models are provided in Supplementary Table S3.

### Cost-effectiveness analysis

Patients treated with ramucirumab plus paclitaxel accrued a total of 1.189 LYs and 0.7281 QALYs, with an estimated total cost of ¥418,087. By contrast, treatment with T-DXd resulted in 1.8835 LYs and 1.1102 QALYs, accompanied by a total cost of ¥650,323.

Compared with the ramucirumab plus paclitaxel regimen, the T-DXd strategy was associated with an incremental cost of ¥232,236 and an additional gain of 0.3821 QALYs. Accordingly, the ICUR for T-DXd versus ramucirumab plus paclitaxel was calculated to be ¥607,788.5 per QALY gained. The results of the cost-effectiveness analysis are summarized in Table 3.

**TABLE 3:** Results of the cost-effectiveness analysis.

Outcomes	Ramucirumab plus paclitaxel regimen	T-DXd regimen
LYs	1.1894	1.8835
Incremental	0.6941	
QALYs	0.7281	1.1102
Incremental	0.3821	
Cost, CNY¥	418,087	650,323
Incremental	232,236	
ICER(¥/LY)	334,585.80	
ICUR(¥/QALY)	607,788.50	

Abbreviation: LY life-year, QALY quality-adjusted life-year, ICER incremental cost-effectiveness ratio, ICUR incremental cost-utility ratio, T-DXd Trastuzumab Deruxtecan

### Sensitivity analysis and additional analysis

**FIGURE 3:**
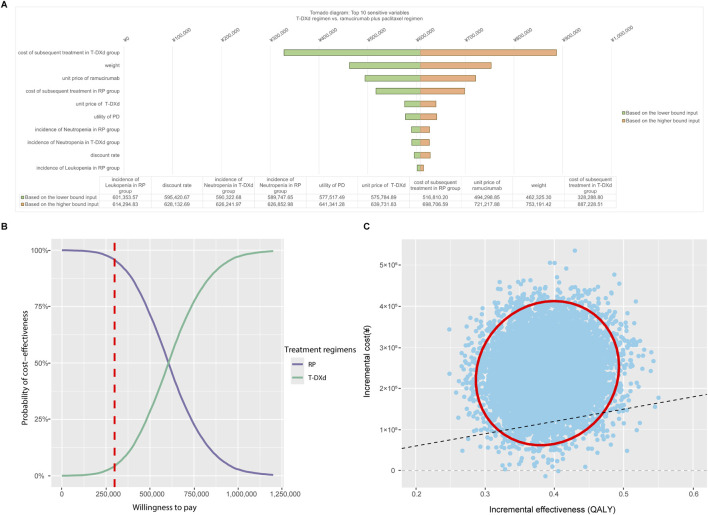
The plot output of the one-way and probabilistic sensitivity analysis. **(A)** The tornado diagram of the ICUR of the T-DXd regimen versus ramucirumab plus paclitaxel regimen; **(B)** The cost-effectiveness acceptable curve of the T-DXd regimen versus ramucirumab plus paclitaxel regimen; **(C)** The incremental cost-effectiveness scatter plot of the T-DXd regimen versus ramucirumab plus paclitaxel regimen.Notes: In the cost-effectiveness acceptable curve, the y-axis indicates the likelihood that a regimen is cost-effective across the willingness-to-pay threshold (x-axis). The red dashed line represents the WTP threshold. The monetary unit of the WTP threshold is Chinese Yuan; In the incremental cost-effectiveness scatter plot, each dot represents one output. The red circle is the 95% confidence ellipse. The black dashed line represents the WTP threshold. Abbreviations: PD progressed disease, T-DXd Trastuzumab Deruxtecan, RP ramucirumab plus paclitaxel, QALY quality-adjusted life-year.

The results of the one-way sensitivity analysis demonstrate the key parameters influencing the robustness of the base-case results. The diagram of tornado (Figure 3A) presented the ten variables with the greatest impact on the ICUR. As can be noticed from the figure, variables including the cost of subsequent treatment in both T-DXd regimen and the ramucirumab plus paclitaxel group, weight, the unit price of ramucirumab and T-DXd exert a significant impact on the ICUR. The ranges of variable alterations, based on the lower bound input and the higher bound input, are clearly visible, varying from ¥ 328,288.80 per QALY to ¥ 887,228.51 per QALY.

The outcomes of the probabilistic sensitivity analysis are illustrated through CEAC (Figure 3B) and incremental cost-effectiveness scatter plots (Figure 3C). At a WTP threshold of ¥298,995 per QALY, the CEAC demonstrates that the probability of the ramucirumab plus paclitaxel regimen being cost-effective was 95.7%, compared with 4.3% for the T-DXd regimen. This suggests that, at the current WTP threshold, ramucirumab plus paclitaxel regimen is more likely to be the preferred strategy. As the WTP threshold increases, the probability of T-DXd being cost-effective rises progressively. When the WTP exceeds ¥607,788.5 per QALY, T-DXd becomes the more favorable option from a cost-effectiveness perspective.

The scatter plot illustrates the distribution of incremental costs and incremental QALYs across simulations. Most simulated outcomes are concentrated within the red oval area and lie above the WTP threshold line, indicating that in the majority of iterations, the incremental cost associated with T-DXd exceeds the acceptable threshold relative to its incremental benefit. These findings are consistent with the CEAC results and support the conclusion that the ramucirumab plus paclitaxel regimen demonstrates greater cost-effectiveness under the current threshold.

In the base-case model, the subsequent treatment strategies essentially reflected a cross-over pattern: patients in the T-DXd arm were assumed to receive the third-line treatment with ramucirumab plus paclitaxel regimen after disease progression, whereas those initially treated with ramucirumab plus paclitaxel were assumed to switch to third-line treatment with T-DXd regimen upon progression. Results from the tornado diagram indicated that the unit price of ramucirumab had a greater impact on the ICUR than the unit price of T-DXd. In addition, the cost of subsequent therapy was identified as a major driver of the ICUR.

**FIGURE 4:**
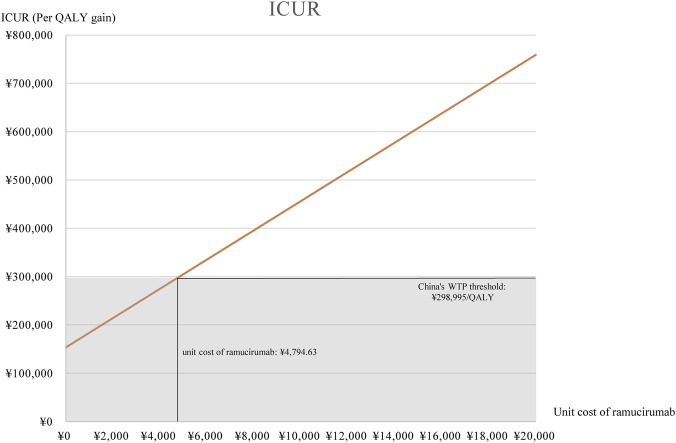
Subsequent treatment Price threshold analysis for T-DXd versus Ramucirumab plus paclitaxel regimen.

To further explore the influence of subsequent treatment on the outcomes, a price-threshold analysis was conducted. In this analysis, the subsequent treatments in both arms were standardized to a common regimen to isolate the effect of subsequent therapy costs. The cost of subsequent treatment in both groups was varied within ¥50,000 to explore its impact on the ICUR. The Figure 4 presented the price-threshold relationship. The results indicated that the ICUR would fall below the predefined WTP threshold when the cycle cost of subsequent treatment was less than ¥28,053. When subsequent treatment costs were standardized within the defined range, T-DXd demonstrated superior cost-effectiveness compared with the ramucirumab plus paclitaxel regimen. Given that several alternative regimens used in later-line settings are associated with lower per-cycle costs, this finding appears clinically and economically plausible.

Notably, the Price threshold figure showed that assuming the cost of subsequent treatment did not exceed ¥9,218 resulted in a negative ICUR. This implies that T-DXd group not only provided greater health benefits but also reduced total costs compared with ramucirumab plus paclitaxel group, thereby representing a dominant strategy under this assumption.

## Discussion

Among patients with HER2-positive metastatic GC or GEJA, the optimal sequencing of HER2-targeted therapies remains an important clinical and policy issue (Cai et al., 2025). Although current Chinese clinical guidelines recommend T-DXd for third-line or later treatment (Chinese Society of Clinical Oncology, 2025), the DESTINY-Gastric04 trial demonstrated superior survival outcomes for T-DXd compared with the standard second-line regimen of ramucirumab plus paclitaxel in HER2-positive GC or GEJA population. Given that both regimens are associated with substantial treatment costs, their incorporation into routine clinical practice raises concerns regarding affordability and resource allocation (Wen et al., 2023).

Owing to the lack of head-to-head clinical studies comparing these two regimens in the past, it has been challenging to quantitatively assess their relative health benefits and economic value. To address this gap, the present study conducted a pharmacoeconomic evaluation based on survival data derived from the DESTINY-Gastric04 trial. From the perspective of the Chinese healthcare system, we assessed the cost-effectiveness of T-DXd compared with the ramucirumab plus paclitaxel regimen as second-line treatment for patients with HER2-positive metastatic GC or GEJA. The findings provide evidence to inform value-based pricing negotiations, reimbursement decision-making, and the efficient allocation of healthcare resources. The current cost-effectiveness analysis indicates that the ICUR of the T-DXd regimen compared with the ramucirumab plus paclitaxel regimen is ¥607,788.5 per QALY, significantly exceeding the predefined WTP threshold. The probabilistic sensitivity analysis further indicated that the probability of the T-DXd regimen being cost-effective at the current WTP threshold was 3.6%. To our knowledge, no previous economic evaluations have directly compared these two treatment strategies in the current settings.

The one-way sensitivity analysis suggested that the unit price of ramucirumab had a greater impact on the ICUR than the price of T-DXd. Firstly, we analyzed the results from a pricing perspective. Although T-DXd has not yet been reimbursed for GC or GEJA, its inclusion in the reimbursement list for HER2-positive breast cancer previously led to substantial price reductions, bringing its cost to a relatively reasonable level. This is further supported by our tornado diagram, which indicates that additional price reductions have a limited impact on the ICUR, with a 25% price decrease lowering the ICUR from ¥607,788.5/QALY to ¥575,784/QALY. In contrast, ramucirumab currently has no reimbursed indications in China and remains relatively expensive, which may substantially influence overall cost-effectiveness results. Notably, although the price of the T-DXd is already relatively low compared with the ramucirumab, the base-case outcome showed that the ramucirumab plus paclitaxel regimen is more cost-effective. This is primarily because the per-cycle cost of ramucirumab is higher than that of T-DXd, and ramucirumab comprises a substantial portion of post-progression treatment costs in the T-DXd arm, thereby exerting a significant impact on the study results. This observation is consistent with the tornado diagram, which highlights the cost of subsequent treatment as a major driver of the ICUR. In the DESTINY-Gastric04 trial, post-progression treatment patterns showed a high proportion of cross-over use: A high proportion of patients in the T-DXd group received third-line treatment with ramucirumab plus paclitaxel after progression, whereas a high proportion of patients initially treated with ramucirumab plus paclitaxel commonly received third-line treatment with T-DXd subsequently. This cross-treatment pattern was reflected in our base-case model assumptions. However, in routine clinical practice, multiple alternative subsequent-line regimens are available, and their costs may vary substantially. To analyze this uncertainty, we conducted a price-threshold analysis in which subsequent treatment in both groups was standardized and varied within a range of ¥0 to ¥50,000. The analysis demonstrated that post-progression treatment costs exerted a pronounced influence on the ICUR. Notably, when the cost of subsequent therapy ranged from ¥0 to ¥9,218, the total treatment cost in the T-DXd arm was lower than ramucirumab plus paclitaxel arm. Under this scenario, T-DXd generated greater health benefits and was associated with lower total costs, indicating economic dominance. When the cost of subsequent therapy ranged from ¥9,218 to ¥28,053, the T-DXd regimen is still a cost-effective option. Furthermore, body weight represents a critical sensitivity factor, as it directly affects the dosing of both T-DXd and ramucirumab, thereby influencing the total treatment costs in each regimen. Given that changes in the cost of ramucirumab have a greater magnitude than those of T-DXd, a lower body weight leads to a relatively greater cost reduction in ramucirumab during subsequent treatment within the T-DXd regimen, ultimately resulting in a lower ICUR. This trend is also reflected in the tornado diagram, which visually illustrates the corresponding increase or decrease in ICUR associated with changes in body weight.

It should be noted that ramucirumab plus paclitaxel remains an important standard second-line therapy for metastatic GC or GEJA when HER2 status is not taken into account. However, in the specific population with HER2-positive disease, the clinical value demonstrated by T-DXd suggests a potentially more favorable therapeutic option, provided that crossover treatments are not applied after disease progression or that subsequent therapy in both arms is standardized to a common regimen. In this context, balancing clinical benefit, drug pricing, and reimbursement policy will therefore be critical for optimizing treatment strategies and ensuring efficient allocation of healthcare resources in China (Zhu Z. et al., 2025).

Our study has several limitations. Health state utility values were not obtained directly from the DESTINY-Gastric04 trial but were extracted from previously published studies. The selected utility estimates were derived from populations similar to the target population of the present analysis, and were measured using the EQ-5D questionnaire survey. Despite being highly relevant, they were still not direct utility data from the DESTINY-Gastric04 trial. As a result, some uncertainty may have been introduced into the model. Secondly, uncertainty was introduced during the reconstruction and extrapolation of survival data. One potential source of bias arises from the transformation of published time-to-survival curves into individual time-to-event data. To address this issue, we applied Guyot’s algorithm, which is widely used in health economic evaluations and has been shown to provide reliable reconstruction of survival data (Saluja et al., 2019). In addition, the choice of parametric survival distribution represents another important source of structural uncertainty. As illustrated in the model fit plots (Supplementary Figure S1), different survival models yielded varying long-term survival projections, which in turn influenced the estimated health benefits. Given the absence of mature long-term follow-up data, this limitation is difficult to eliminate entirely. In this study, we selected the most appropriate distributions based on statistical criteria, including AIC and BIC values, together with visual inspection of the fitted curves. Thirdly, a major limitation lies in the simplified assumption adopted for subsequent treatment strategies. As shown in Supplementary Table S2, more than 60 post-progression treatment regimens were reported in the DESTINY-Gastric04 trial, including multiple combination regimens and third-line or later-line therapies. Because fully reconstructing all possible treatment sequences would substantially increase model complexity and structural uncertainty, the present analysis used the most frequently administered regimens to approximate post-progression treatment patterns. Although this approach improved model feasibility and preserved the major clinical characteristics of the trial population, it may not fully capture the heterogeneity of real-world treatment pathways. Finally, our study did not carry out a subgroup analysis. Although the DESTINY-Gastric04 trial provided HR values of PFS and OS stages in different subgroups, the method of survival simulation by curve extrapolation requires survival curves or individual patient data for different subgroups. The lack of such data restricted our ability to perform subgroup analysis. Many scholars estimated subgroup analysis results by changing HR values. This method they applied was based on the assumption of the control group’s survival curves in the overall population rather than survival curves of the subgroup population. Therefore, we did not adopt this assumption-based approach.

## Conclusion

In conclusion, from the perspective of the Chinese healthcare system, T-DXd was not cost-effective compared with the ramucirumab plus paclitaxel regimen for HER2-positive metastatic GC or GEJA under the post-progression treatment patterns observed in the clinical trial. When subsequent treatment strategies were standardized to a common regimen between groups and the cycle cost of subsequent treatment was limited to ¥28,053 or less, T-DXd became the more cost-effective option. Furthermore, when the analysis was restricted to the pre-progression phase, T-DXd was associated with lower total costs and greater survival benefits, indicating a cost-effective strategy in this setting.

## Data Availability

The original contributions presented in the study are included in the article/Supplementary Material, further inquiries can be directed to the corresponding author.

## Supplementary material

SUPPLEMENTARY FIGURE S1 The fit figures based on other alternative distributions. **Abbreviations: *T-DXd*** Trastuzumab Deruxtecan, ***RP*** ramucirumab plus paclitaxel. Notes: Each cycle of the x-axis is four weeks.

SUPPLEMENTARY FIGURE S2 Price threshold analysis of ramucirumab unit cost on its impact on ICUR.
