# Supplementary material for: Cost-effectiveness analysis of trastuzumab deruxtecan versus ramucirumab plus paclitaxel as second-line treatment for HER2-positive metastatic gastric cancer or gastroesophageal junction adenocarcinoma
Source: Front Pharmacol. 2026 Jun 25;17:1811077. doi: 10.3389/fphar.2026.1811077 (PMC13345832; doi:10.3389/fphar.2026.1811077)
Supplement: Supplementary file 1 [file Supplementaryfile1.docx]

| Supplementary Table 1 The CHEERS 2022 checklist | | | |
| --- | --- | --- | --- |
| Section/topic | **Item No** | **Guidance for reporting** | **Article section** |
| Title | | | |
| Title | 1 | Identify the study as an economic evaluation and specify the interventions being compared. | Title |
| Abstract | | | |
| Abstract | 2 | Provide a structured summary that highlights context, key methods, results, and alternative analyses. | Abstract |
| Introduction | | | |
| Background and objectives | 3 | Give the context for the study, the study question, and its practical relevance for decision making in policy or practice. | Introduction |
| Methods | | | |
| Health economic analysis plan | 4 | Indicate whether a health economic analysis plan was developed and where available. | Materials and Methods |
| Study population | 5 | Describe characteristics of the study population (such as age range, demographics, socioeconomic, or clinical characteristics). | Materials and Methods- Clinical data |
| Setting and location | 6 | Provide relevant contextual information that may influence findings. | Materials and Methods |
| Comparators | 7 | Describe the interventions or strategies being compared and why chosen. | Materials and Methods- Treatment regimens and Resource use |
| Perspective | 8 | State the perspective(s) adopted by the study and why chosen. | Materials and Methods- Model structure |
| Time horizon | 9 | State the time horizon for the study and why appropriate. | Materials and Methods- Model structure |
| Discount rate | 10 | Report the discount rate(s) and reason chosen. | Materials and Methods-Analyses |
| Selection of outcomes | 11 | Describe what outcomes were used as the measure(s) of benefit(s) and harm(s). | Materials and Methods-Analyses |
| Measurement of outcomes | 12 | Describe how outcomes used to capture benefit(s) and harm(s) were measured. | Materials and Methods- Model structure, Analyses |
| Valuation of outcomes | 13 | Describe the population and methods used to measure and value outcomes. | Materials and Methods- Model structure |
| Measurement and valuation of resources and costs | 14 | Describe how costs were valued. | Materials and Methods-Costs and utilities |
| Currency, price date, and conversion | 15 | Report the dates of the estimated resource quantities and unit costs, plus the currency and year of conversion. | Materials and Methods-Costs and utilities |
| Rationale and description of model | 16 | If modelling is used, describe in detail and why used. Report if the model is publicly available and where it can be accessed. | Materials and Methods- Model structure |
| Analytics and assumptions | 17 | Describe any methods for analysing or statistically transforming data, any extrapolation methods, and approaches for validating any model used. | Materials and Methods-Clinical data |
| Characterizing heterogeneity | 18 | Describe any methods used for estimating how the results of the study vary for subgroups. | Not applicable |
| Characterizing distributional effects | 19 | Describe how impacts are distributed across different individuals or adjustments made to reflect priority populations. | Not applicable |
| Characterizing uncertainty | 20 | Describe methods to characterise any sources of uncertainty in the analysis. | Materials and Methods-Analyses |
| Approach to engagement with patients and others affected by the study | 21 | Describe any approaches to engage patients or service recipients, the general public, communities, or stakeholders (such as clinicians or payers) in the design of the study. | Not applicable |
| Results | | | |
| Study parameters | 22 | Report all analytic inputs (such as values, ranges, references) including uncertainty or distributional assumptions. | Table 1 and Table 2 |
| Summary of main results | 23 | Report the mean values for the main categories of costs and outcomes of interest and summarise them in the most appropriate overall measure. | Results-Cost-effectiveness analysis; Table 3 |
| Effect of uncertainty | 24 | Describe how uncertainty about analytic judgments, inputs, or projections affect findings. Report the effect of choice of discount rate and time horizon, if applicable. | Results-Sensitivity analysis and additional analysis;  Figure 3 |
| Effect of engagement with patients and others affected by the study | 25 | Report on any difference patient/service recipient, general public, community, or stakeholder involvement made to the approach or findings of the study | Not applicable |
| Discussion | | | |
| Study findings, limitations, generalizability, and current knowledge | 26 | Report key findings, limitations, ethical or equity considerations not captured, and how these could affect patients, policy, or practice. | Discussion |
| Other relevant information | | | |
| Source of funding | 27 | Describe how the study was funded and any role of the funder in the identification, design, conduct, and reporting of the analysis | Declarations- Funding |
| Conflicts of interest | 28 | Report authors conflicts of interest according to journal or International Committee of Medical Journal Editors requirements. | Declarations- Conflict of interest statement: |
| For consistency, the CHEERS Statement checklist format is based on the format of the CONSORT 2 statement checklist^1^ | | | |

| **Supplementary Table 2: Post-trial therapy regimens** | | |
| --- | --- | --- |
| **no. (%)** | **T-DXd**  **n = 246** | **RP n = 248** |
| BDC 1001 | 0 | 1 (0.4) |
| Bevacizumab | 0 | 1 (0.4) |
| *Brucea javanica* oil | 0 | 1 (0.4) |
| Calcineurin subunit B | 0 | 1 (0.4) |
| Calcium folinate | 5 (2.0) | 2 (0.8) |
| Calcium folinate; fluorouracil; irinotecan | 1 (0.4) | 0 (0.0) |
| Calcium folinate; fluorouracil; irinotecan hydrochloride | 11 (4.5) | 12 (4.8) |
| Capecitabine | 7 (2.8) | 0 |
| Capecitabine; cisplatin | 1 (0.4) | 0 |
| Capecitabine; irinotecan | 0 | 1 (0.4) |
| Capecitabine; oxaliplatin | 1 (0.4) | 1 (0.4) |
| Carboplatin | 1 (0.4) | 0 |
| Cisplatin | 1 (0.4) | 2 (0.8) |
| Combinations of antineoplastic agents | 1 (0.4) | 0 |
| Disitamab vedotin | 5 (2.0) | 12 (4.8) |
| Docetaxel | 4 (1.6) | 4 (1.6) |
| Fluorouracil | 10 (4.1) | 11 (4.4) |
| Fluorouracil sodium | 1 (0.4) | 0 |
| Fluorouracil; folinic acid; oxaliplatin | 2 (0.8) | 3 (1.2) |
| Fluorouracil; irinotecan | 0 | 1 (0.4) |
| Fluorouracil; oxaliplatin | 1 (0.4) | 0 |
| Folinic acid | 2 (0.8) | 4 (1.6) |
| Fruquintinib | 3 (1.2) | 1 (0.4) |
| Gimeracil; oteracil potassium; tegafur | 3 (1.2) | 0 |
| GS 1811 | 0 | 1 (0.4) |
| Immunotherapy | 1 (0.4) | 1 (0.4) |
| Investigational drug | 5 (2.0) | 10 (4.0) |
| Irinotecan | 16 (6.5) | 16 (6.5) |
| Irinotecan hydrochloride | 2 (0.8) | 7 (2.8) |
| Irinotecan hydrochloride trihydrate | 1 (0.4) | 0 |
| Irinotecan hydrochloride trihydrate liposomal | 1 (0.4) | 0 |
| Levofolinic acid | 1 (0.4) | 0 |
| Levonorgestrel | 0 | 1 (0.4) |
| *Marsdenia tenacissima* stem | 0 | 1 (0.4) |
| Nivolumab | 14 (5.7) | 14 (5.6) |
| Oxaliplatin | 10 (4.1) | 1 (0.4) |
| Oxyfedrine hydrochloride | 0 | 1 (0.4) |
| Paclitaxel | 63 (25.6) | 8 (3.2) |
| Paclitaxel nanoparticle albumin-bound | 13 (5.3) | 0 |
| Paclitaxel; ramucirumab | 13 (5.3) | 3 (1.2) |
| Pembrolizumab | 2 (0.8) | 2 (0.8) |
| Pyrotinib maleate | 1 (0.4) | 1 (0.4) |
| Ramucirumab | 43 (17.5) | 8 (3.2) |
| Regorafenib | 0 | 1 (0.4) |
| Rivoceranib | 1 (0.4) | 1 (0.4) |
| Rivoceranib mesylate | 1 (0.4) | 2 (0.8) |
| Runimotamab | 0 | 1 (0.4) |
| Serplulimab | 0 | 1 (0.4) |
| Sintilimab | 5 (2.0) | 2 (0.8) |
| Sulfasalazine | 0 | 1 (0.4) |
| Tegafur | 0 | 1 (0.4) |
| Tipiracil | 1 (0.4) | 0 |
| Tipiracil hydrochloride; trifluridine | 11 (4.5) | 11 (4.4) |
| Tipiracil; trifluridine | 2 (0.8) | 1 (0.4) |
| Tislelizumab | 0 | 1 (0.4) |
| Toripalimab | 0 | 1 (0.4) |
| Trastuzumab | 12 (4.9) | 8 (3.2) |
| Trastuzumab deruxtecan | 3 (1.2) | 52 (21.0) |
| Trastuzumab mafodotin | 0 | 1 (0.4) |
| Trifluridine | 2 (0.8) | 2 (0.8) |
| Tumor necrosis factor NOS | 1 (0.4) | 0 |
| Zimberelimab | 0 | 1 (0.4) |
| Patients may have been treated with more than 1 type of posttrial anticancer therapy.  **Abbreviations:** ***T-DXd*** Trastuzumab Deruxtecan, ***RP*** ramucirumab plus paclitaxel. | | |

| **Supplementary Table 3. Detailed fitting curve parameters** | | | | | | | | |
| --- | --- | --- | --- | --- | --- | --- | --- | --- |
| Treatment arm | Distribution | Shape parameters | Value | L95% | U95% | SE | AIC | BIC |
| PFS: T-DXd regimen | Log-logistic | shape | 2.093 | 1.847 | 2.372 | 0.134 | 1042.023 | 1049.034 |
|  |  | scale | 7.026 | 6.270 | 7.872 | 0.408 |  |  |
|  | Weibull | shape | 1.362 | 1.216 | 1.526 | 0.079 | 1072.201 | 1079.212 |
|  |  | scale | 10.367 | 9.261 | 11.606 | 0.597 |  |  |
|  | Exponential | rate | 0.09536 | 0.08179 | 0.11118 | 0.00747 | 1094.139 | 1097.645 |
|  | Lognormal | meanlog | 1.9646 | 1.8487 | 2.0805 | 0.0591 | 1040.517 | 1047.527 |
|  |  | sdlog | 0.8286 | 0.7421 | 0.9253 | 0.0466 |  |  |
|  | Gompertz | shape | 0.01978 | -0.00563 | 0.04518 | 0.01296 | 1093.951 | 1100.962 |
|  |  | rate | 0.08455 | 0.06758 | 0.10578 | 0.00966 |  |  |
| PFS: RP regimen | Log-logistic | shape | 2.099 | 1.849 | 2.382 | 0.136 | 938.5868 | 945.6137 |
|  |  | scale | 5.661 | 5.044 | 6.355 | 0.334 |  |  |
|  | Weibull | shape | 1.405 | 1.257 | 1.571 | 0.08 | 952.772 | 959.7989 |
|  |  | scale | 8.037 | 7.191 | 8.982 | 0.456 |  |  |
|  | Exponential | rate | 0.12306 | 0.10529 | 0.14382 | 0.00979 | 980.0571 | 983.5705 |
|  | Lognormal | meanlog | 1.7149 | 1.5921 | 1.8376 | 0.0626 | 943.9427 | 950.9695 |
|  |  | sdlog | 0.8590 | 0.7699 | 0.9584 | 0.0480 |  |  |
|  | Gompertz | shape | 0.03557 | 0.00822 | 0.06293 | 0.01396 | 976.3839 | 983.4108 |
|  |  | rate | 0.10319 | 0.08317 | 0.12805 | 0.01136 |  |  |
| OS: T-DXd regimen | Log-logistic | shape | 1.782 | 1.541 | 2.062 | 0.132 | 1028.415 | 1035.426 |
|  |  | scale | 15.828 | 13.745 | 18.227 | 1.140 |  |  |
|  | Weibull | shape | 1.398 | 1.213 | 1.612 | 0.101 | 1033.453 | 1040.464 |
|  |  | scale | 21.647 | 19.014 | 24.644 | 1.432 |  |  |
|  | Exponential | rate | 0.04113 | 0.03451 | 0.04901 | 0.00368 | 1049.78 | 1053.285 |
|  | Lognormal | meanlog | 2.7740 | 2.6182 | 2.9297 | 0.0795 | 1032.176 | 1039.186 |
|  |  | sdlog | 1.0130 | 0.8897 | 1.1534 | 0.0671 |  |  |
|  | Gompertz | shape | 0.03195 | 0.00967 | 0.05423 | 0.01137 | 1044.401 | 1051.411 |
|  |  | rate | 0.03027 | 0.02259 | 0.04057 | 0.00452 |  |  |
| OS: RP regimen | Log-logistic | shape | 1.756 | 1.536 | 2.007 | 0.120 | 1100.601 | 1107.628 |
|  |  | scale | 12.262 | 10.673 | 14.088 | 0.868 |  |  |
|  | Weibull | shape | 1.3841 | 1.2166 | 1.5746 | 0.0911 | 1091.773 | 1098.8 |
|  |  | scale | 16.7342 | 14.8575 | 18.8479 | 1.0156 |  |  |
|  | Exponential | rate | 0.05633 | 0.04782 | 0.06636 | 0.00471 | 1110.675 | 1114.189 |
|  | Lognormal | meanlog | 2.4851 | 2.3305 | 2.6398 | 0.0789 | 1111.774 | 1118.801 |
|  |  | sdlog | 1.0564 | 0.9393 | 1.1880 | 0.0633 |  |  |
|  | Gompertz | shape | 0.04528 | 0.02454 | 0.06601 | 0.01058 | 1096.334 | 1103.361 |
|  |  | rate | 0.03746 | 0.02863 | 0.04901 | 0.00514 |  |  |

**Abbreviations:** ***T-DXd*** Trastuzumab Deruxtecan, ***RP*** ramucirumab plus paclitaxel, ***PFS*** progression-free survival, ***OS*** overall survival.

| Supplementary Table 4. Scenario analysis including pre-treatment endoscopy and biopsy costs | | | | |
| --- | --- | --- | --- | --- |
| Regimen | **QALYs** | **Cost, CNY** **¥** | **ICUR(¥/QALY)** | **Preferred strategy** |
| T-DXd | 1.1102 | 651,487.18 | 607,788.5 | RP |
| RP | 0.7281 | 419,251.18 |  |  |
| Abbreviations: *T-DXd* Trastuzumab Deruxtecan, *RP* Ramucirumab plus paclitaxel, *LY* life-year, *QALY* quality-adjusted life-year, *ICUR* incremental cost-utility ratio. | | | | |


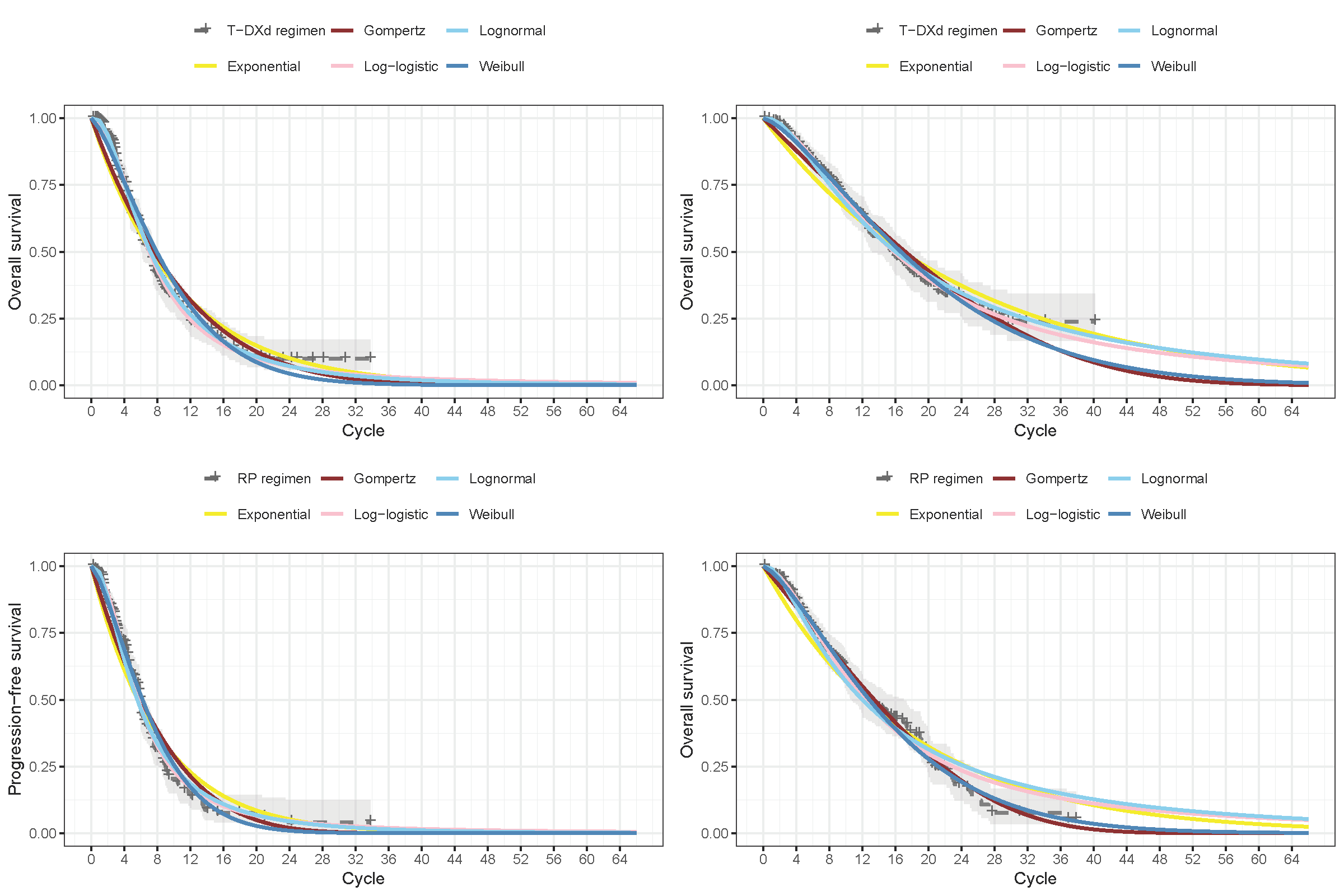


**Supplementary Figure 1**. The fit figures based on other alternative distributions.

**Abbreviations:** ***T-DXd*** Trastuzumab Deruxtecan, ***RP*** ramucirumab plus paclitaxel. Notes: Each cycle of the x-axis is four weeks.


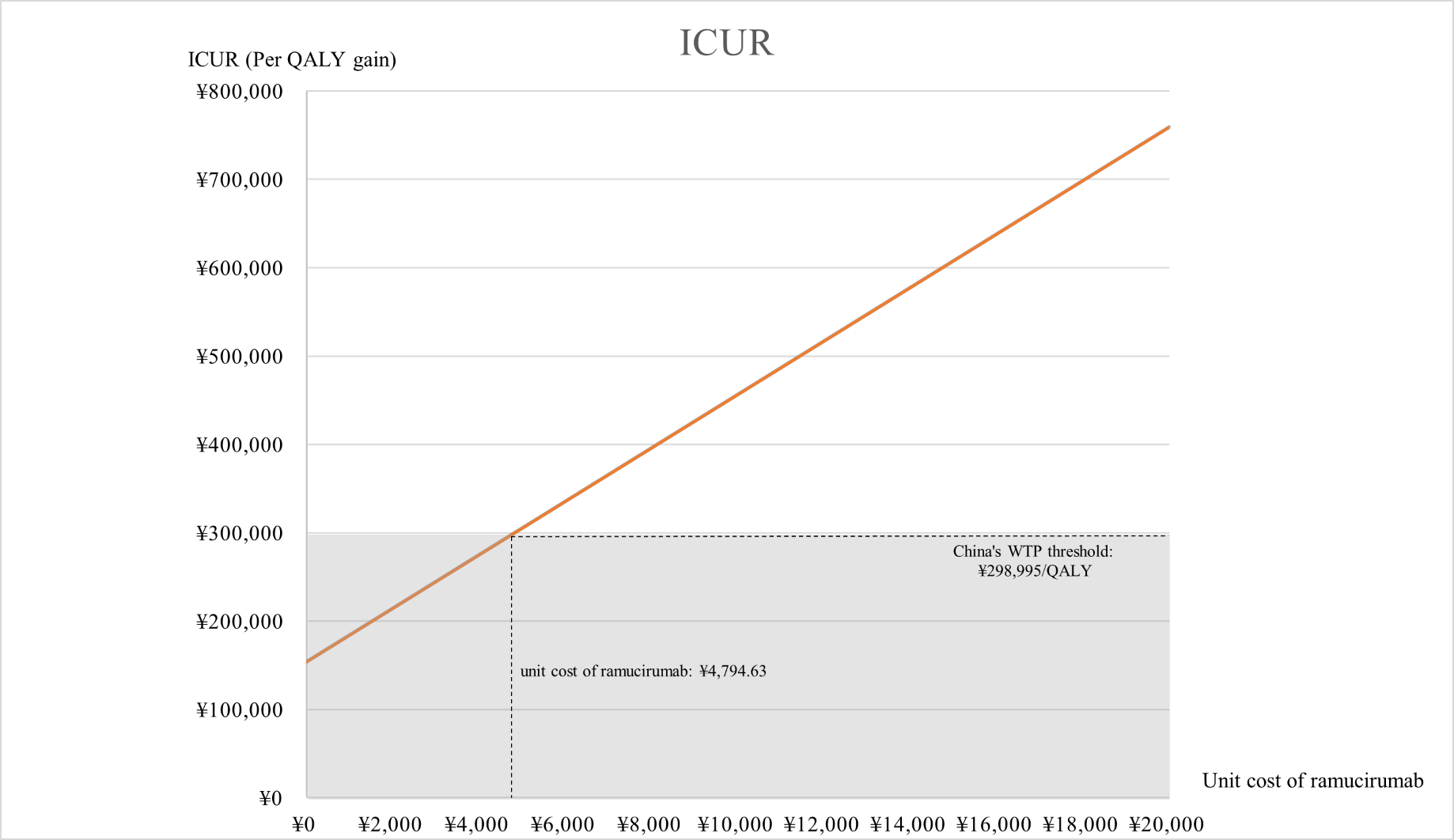


**Supplementary Figure 2.** Price threshold analysis of ramucirumab unit cost on its impact on ICUR.
